# Association between Recommendations from Public Health Nurses, Medical Professionals, and Family Members and Participation in Health Checkups among Middle-aged Community Residents with National Health Insurance

**DOI:** 10.31662/jmaj.2021-0225

**Published:** 2022-03-18

**Authors:** Nao Sonoda, Chie Koh, Risa Yasumoto, Meng Li, Kaori Watanabe, Chikage Tsuzuki, Akiko Morimoto

**Affiliations:** 1 Graduate School of Nursing, Osaka Prefecture University, Osaka, Japan

**Keywords:** health checkups, recommendation, public health nurses, medical professionals, middle-aged community residents

## Abstract

**Introduction:**

Participation in specific health checkups is low in Japan, especially among middle-aged community residents with municipal National Health Insurance (NHI). This study explored associations between recommendations from public health nurses, medical professionals, and family members and participation in specific health checkups among middle-aged Japanese community residents with NHI.

**Methods:**

This mail survey was conducted in 2020, and it included 33,902 community residents with NHI aged 40-64 years from five cities in Osaka Prefecture, Japan. Of these, 12,446 (36.7%) community residents agreed to participate in the survey. After excluding those with missing data, 11,180 participants were included in the analyses. Participants were classified into a participation group and a nonparticipation group. Those who selected “I underwent a specific health checkup in the past year” were classified as the participation group.

**Results:**

Of the 11,180 community residents with NHI, 4,384 (39.2%) were classified in the participation group. After adjusting for confounding factors, the presence (vs. absence) of recommendations from public health nurses (multivariable-adjusted odds ratio [OR], 1.81; 95% confidence interval [CI], 1.47-2.24), primary care physicians (multivariable-adjusted OR, 2.79; 95% CI, 2.49-3.13), nurses (multivariable-adjusted OR, 2.06; 95% CI, 1.57-2.69), and family members (multivariable-adjusted OR, 1.22; 95% CI, 1.12-1.32) was positively associated with participation in specific health checkups.

**Conclusions:**

Our findings suggest that recommendations from public health nurses, medical professionals (primary care physicians and nurses), and family members may be important to promote participation in specific health checkups among middle-aged Japanese community residents with NHI.

## Introduction

In Japan, all medical insurers are obliged to conduct annual specific health checkups. These checkups are a part of the National Health Service system, target all insured people aged 40-74 years, and aim to facilitate prevention and early detection of lifestyle-related diseases such as diabetes, hypertension, and dyslipidemia ^(1)^. Previous studies suggested that participation in health checkups was associated with lower mortality ^(2), (3), (4), (5)^, and all insured persons aged 40-74 years are required to undergo a specific health checkup every year ^(6)^. However, the participation rate in specific health checkups is low in Japan, especially among middle-aged community residents with municipal National Health Insurance (NHI) (participation rate: 38.0%) ^(7)^. Improving the participation rate in this population is an important issue.

Clarifying factors associated with participation in health checkups among middle-aged community residents with NHI could be useful in determining appropriate interventions to promote participation. Previous studies suggested that recommendations from primary care physicians and family members were positively associated with participation in cancer screening ^(8), (9), (10)^. However, regarding specific health checkups, although previous studies have shown that personal factors, such as sex, education level, subjective economic status, and living with someone, were associated with participation in health checkups ^(11), (12), (13)^, the association between recommendations from public health nurses, medical professionals, and family members and participation in specific health checkups has not been evaluated. Therefore, we assessed the associations between recommendations from public health nurses, medical professionals (primary care physicians and nurses), and family members and participation in specific health checkups among middle-aged Japanese community residents with NHI.

## Materials and Methods

### Study participants and procedure

Medical insurance in Japan is roughly divided into two groups: community-based and employee-based. Municipal NHI is a representative of community-based medical insurance. This is managed by local municipalities and includes the largest group of insured people, mainly comprising self-employed workers, part-time workers, farmers, homemakers, unemployed people, and retired people.

A mail survey that involved all community residents with NHI aged 40-64 years in five cities in three areas of Osaka Prefecture, Japan, was conducted in 2020. This survey included 33,902 community residents with NHI aged 40-64 years. A reminder postcard was mailed one week after the self-administered questionnaire was mailed to participants. In total, 12,446 (36.7%) community residents agreed to participate in the mail survey. After excluding those with missing data, 11,180 community residents with NHI aged 40-64 years were included in the analyses.

The study protocol was prepared in accordance with the Declaration of Helsinki and approved by the Institutional Review Boards of Osaka Prefecture University (approval date October 5, 2020; approval no. 2020-28). Informed consent was obtained from all participants who were included in this study.

### Outcome

Information on participation in specific health checkups was obtained using a self-administered questionnaire. Study participants were classified into two groups: a participation group and a nonparticipation group. Those who selected “I underwent a specific health checkup in the past year” were classified as the participation group.

### Exposure variables and potential confounding factors

Information obtained using the self-administered questionnaire included the following: the presence or absence of recommendations from public health nurses, medical professionals (primary care physicians and nurses), and family members to participate in specific health checkups in the past year; age; sex; education level (≤12 or >12 years); subjective economic status (very good plus good, average, or poor plus very poor); living with someone (yes or no); occupation (self-employed workers, part-time workers, plus farmers, homemakers, or unemployed plus retired); stages of health behavior change (precontemplation, contemplation plus preparation, or action plus maintenance); drinking status (regular drinkers or occasional and nondrinkers); smoking status (current smokers, ex-smokers, or nonsmokers); body mass index (BMI); regular visits to medical institutions (presence or absence); and the number of visits to medical institutions. BMI was calculated as weight (kg) divided by height in meters squared (m^2^).

### Statistical analyses

Differences in study variables between the participation and nonparticipation groups were determined using t-tests for continuous data with a normal distribution, Mann-Whitney U tests for continuous data with a nonnormal distribution, and chi-square tests for dichotomous and categorical data.

The proportions of those in the participation group by recommendations from public health nurses, medical professionals (primary care physicians and nurses), and family members were compared using chi-square tests. Logistic regression models were used to estimate age- and multivariable-adjusted odds ratios (ORs) and 95% confidence intervals (CIs) for participation in specific health checkups (response variable: 1 = participation group; 0 = nonparticipation group) by recommendations from public health nurses, medical professionals, and family members. Age, sex, education level (≤12 or >12 years), subjective economic status (very good plus good, average, or poor plus very poor), occupation (self-employed workers, part-time workers, plus farmers, homemakers, or unemployed plus retired), stages of health behavior change (precontemplation, contemplation plus preparation, or action plus maintenance), smoking status (current smokers, ex-smokers, or nonsmokers), BMI (≥25.0 or <25.0 kg/m^2^), and regular visits to medical institutions (presence or absence) were included in Model 1. All factors in Model 1 as well as recommendations from public health nurses, medical professionals, and family members were included in Model 2. Recommendations from primary care physicians and nurses were not included in the same model because they were strongly related. Furthermore, the analysis was repeated after the participants were stratified by stages of health behavior change and again after the participants were stratified by regular visits to medical institutions.

All data were analyzed using SPSS statistical software version 26 (IBM SPSS Japan, Tokyo, Japan). All reported *p*-values were two-tailed, and values <0.05 were considered statistically significant.

## Results

In this study, 4,384 (39.2%) participants were classified in the participation group, and 6,796 (60.8%) were classified in the nonparticipation group. Table 1 shows the differences in study variables between the participation and nonparticipation groups. Age (p < 0.001), sex (p < 0.001), education level (p < 0.001), subjective economic status (p < 0.001), occupation (p < 0.001), stages of health behavior change (p < 0.001), smoking status (p < 0.001), BMI (p < 0.001), regular visits to medical institutions (p < 0.001), and the number of visits to medical institutions (p < 0.001) differed significantly between the two groups.

**Table 1.** Differences in Study Variables between the Participation and Nonparticipation Groups.

Factors	Participation group	Nonparticipation group	*p*-value
n	4,384	6,796
Age (years)	55.3 (6.8)	54.2 (6.7)	<0.001
Men, %	37.4	41.8	<0.001
Education level: ≤12 years, %	47.8	53.6	<0.001
Subjective economic status, %			<0.001
Very good and good	25.7	17.6
Average	23.9	21.7
Poor and very poor	50.4	60.6
Living with someone: yes, %	86.6	85.4	0.093
Occupation, %			<0.001
Self-employed workers, part-time workers, and farmers	65.2	64.0
Homemakers	19.1	15.1
Unemployed/retired	15.6	20.9
Stages of health behavior change, %			<0.001
Precontemplation	22.7	27.9
Contemplation/preparation	45.7	46.0
Action/maintenance	31.7	26.0
Drinking status: regular drinkers, %	24.9	24.0	0.240
Smoking status, %			<0.001
Current smokers	17.4	24.4
Ex-smokers	21.9	20.8
Nonsmokers	60.7	54.8
Body mass index: ≥25.0 kg/m^2^, %	24.2	30.1	<0.001
Regular visits to medical institutions: presence, %	73.4	63.2	<0.001
Number of visits to medical institutions (times/year)	6.0 (2.0, 12.0)	4.0 (1.0, 12.0)	<0.001

Continuous data with a normal distribution were analyzed with t-tests and shown as mean (standard deviation). Continuous data with a nonnormal distribution were analyzed with Mann-Whitney U tests and shown as median (25th and 75th percentiles). Dichotomous and categorical data were analyzed with chi-square tests and shown as %. Those who selected “I underwent a specific health checkup in the past year” were classified as the participation group.

Table 2 shows multivariable-adjusted ORs and 95% CIs for participation in specific health checkups by recommendations from public health nurses, medical professionals (primary care physicians and nurses), and family members among middle-aged community residents with NHI. The analysis of the participation group showed the following: 56.3% of participants had received recommendations from public health nurses and 38.6% had not (p < 0.001); 63.0% had received recommendations from a primary care physician and 35.2% had not (p < 0.001); 59.1% had received recommendations from nurses and 38.8% had not (p < 0.001); and 42.6% had received recommendations from family members and 37.2% had not (p < 0.001). After adjusting for confounding factors, the presence (vs. absence) of recommendations from public health nurses (multivariable-adjusted OR, 1.81; 95% CI, 1.47-2.24), primary care physicians (multivariable-adjusted OR, 2.79; 95% CI, 2.49-3.13), nurses (multivariable-adjusted OR, 2.06; 95% CI, 1.57-2.69), and family members (multivariable-adjusted OR, 1.22; 95% CI, 1.12-1.32) was all positively associated with participation in specific health checkups.

**Table 2.** Multivariable-adjusted Odds Ratios and 95% Confidence Intervals for Participation in Specific Health Checkups by Recommendation from Public Health Nurses, Primary Care Physicians, Nurses, and Family Members among Middle-aged Community Residents with National Health Insurance.

Recommendation from public health nurses, medical professionals, and family members to participate in specific health checkups in the past year	Comparison	Proportions of those in the participation group, % (case/n)	Age-adjusted OR (95% CI) for participation in specific health checkups	Multivariable-adjusted OR (95% CI) for participation in specific health checkups	
				Model 1^*^	Model 2
Municipality				
Public health nurses	Absence	38.6 (4,160/10,782)	1.0	1.0	1.0
	Presence	56.3 (224/398)	2.00 (1.63-2.45)	2.02 (1.65-2.49)	1.81 (1.47-2.24)^**^
		*p* <0.001
Medical institution
Primary care physicians	Absence	35.2 (3,359/9,553)	1.0	1.0	1.0
	Presence	63.0 (1,025/1,627)	3.02 (2.71-3.37)	2.91 (2.60-3.26)	2.79 (2.49-3.13)^***^
		*p* <0.001
Nurses	Absence	38.8 (4,241/10,938)	1.0	1.0	1.0
	Presence	59.1 (143/242)	2.29 (1.77-2.97)	2.29 (1.75-2.98)	2.06 (1.57-2.69)^***^
		*p* < 0.001
Home
Family members	Absence	37.2 (2,604/7,002)	1.0	1.0	1.0
	Presence	42.6 (1,780/4,178)	1.30 (1.20-1.41)	1.32 (1.22-1.43)	1.22 (1.12-1.32)^****^
		*p* <0.001

Those who selected “I underwent a specific health checkup in the past year” were classified as the participation group. Response variable: 1 = participation group and 0 = nonparticipation group. ^*^Model 1: Adjusted for age, sex, education level (≤12 or >12 years), subjective economic status (very good plus good, average, or poor plus very poor), occupation (self-employed workers, part-time workers, plus farmers, homemakers, or unemployed plus retired), stages of health behavior change (precontemplation, contemplation plus preparation, or action plus maintenance), smoking status (current smokers, ex-smokers, or nonsmokers), body mass index (≥25.0 or <25.0 kg/m^2^), and regular visits to medical institutions (presence or absence). ^**^Adjusted for all factors in Model 1 plus recommendation from primary care physicians (presence or absence) and recommendation from family members (presence or absence). ^***^Adjusted for all factors in Model 1 plus recommendation from public health nurses (presence or absence) and recommendation from family members (presence or absence). ^****^Adjusted for all factors in Model 1 plus recommendation from public health nurses (presence or absence) and recommendation from primary care physicians (presence or absence). OR, odds ratio; CI, confidence interval.

Table 3 shows the analysis stratified by health behavior change stages. After adjusting for confounding factors, the presence of recommendations from public health nurses, primary care physicians, and family members was positively associated with participation in health checkups among participants in the precontemplation, contemplation plus preparation, and action plus maintenance stages. After adjusting for confounding factors, the presence of recommendations from nurses was positively associated with participation in health checkups among those in the contemplation plus preparation and action plus maintenance stages. Table 4 shows the analysis stratified by regular visits to medical institutions. After adjusting for confounding factors, the presence of recommendations from public health nurses, primary care physicians, nurses, and family members was all positively associated with participation in health checkups among community residents with and without regular visits to medical institutions.

**Table 3.** Multivariable-adjusted Odds Ratios and 95% Confidence Intervals for Participation in Specific Health Checkups by Recommendations from Public Health Nurses, Primary Care Physicians, Nurses, and Family Members among Middle-aged Community Residents with National Health Insurance: Stratified Analysis of Health Behavior Change Stages.

Recommendation from public health nurses, medical professionals, and family members to participate in specific health checkups in the past year	Comparison	Proportions of those in the participation group, % (case/n)	Age-adjusted OR (95% CI) for participation in specific health checkups	Multivariable-adjusted OR (95% CI) for participation in specific health checkups	
				Model 1^*^	Model 2
Community residents in the precontemplation stage (n = 2,892)					
Public health nurses	Absence	33.9 (957/2,823)	1.0	1.0	1.0
	Presence	53.6 (37/69)	2.19 (1.35-3.54)	2.16 (1.32-3.54)	2.07 (1.26-3.41)^**^
Primary care physicians	Absence	31.3 (815/2,603)	1.0	1.0	1.0
	Presence	61.9 (179/289)	3.44 (2.67-4.43)	3.29 (2.53-4.27)	3.19 (2.45-4.15)^***^
Nurses	Absence	34.3 (980/2,860)	1.0	1.0	1.0
	Presence	43.8 (14/32)	1.52 (0.75-3.08)	1.59 (0.77-3.30)	1.47 (0.71-3.04)^***^
Family members	Absence	32.5 (624/1,919)	1.0	1.0	1.0
	Presence	38.0 (370/973)	1.32 (1.13-1.56)	1.34 (1.14-1.59)	1.27 (1.07-1.51)^****^
Community residents in the contemplation and preparation stages (n = 5,131)					
Public health nurses	Absence	38.5 (1,899/4,935)	1.0	1.0	1.0
	Presence	52.6 (103/196)	1.71 (1.28-2.28)	1.80 (1.34-2.41)	1.55 (1.15-2.10)^**^
Primary care physicians	Absence	34.6 (1,491/4,309)	1.0	1.0	1.0
	Presence	62.2 (511/822)	2.96 (2.53-3.46)	2.89 (2.46-3.39)	2.79 (2.37-3.28)^***^
Nurses	Absence	38.5 (1,934/5,017)	1.0	1.0	1.0
	Presence	59.6 (68/114)	2.37 (1.62-3.47)	2.44 (1.66-3.59)	2.22 (1.51-3.28)^***^
Family members	Absence	37.5 (1,139/3,038)	1.0	1.0	1.0
	Presence	41.2 (863/2,093)	1.21 (1.08-1.36)	1.25 (1.11-1.40)	1.15 (1.02-1.30)^****^
Community residents in the action and maintenance stages (n = 3,157)					
Public health nurses	Absence	43.1 (1,304/3,024)	1.0	1.0	1.0
	Presence	63.2 (84/133)	2.24 (1.56-3.21)	2.30 (1.60-3.31)	2.11 (1.45-3.07)^**^
Primary care physicians	Absence	39.9 (1,053/2,641)	1.0	1.0	1.0
	Presence	64.9 (335/516)	2.72 (2.24-3.32)	2.75 (2.25-3.37)	2.59 (2.11-3.18)^***^
Nurses	Absence	43.4 (1,327/3,061)	1.0	1.0	1.0
	Presence	63.5 (61/96)	2.28 (1.49-3.48)	2.43 (1.58-3.74)	2.14 (1.38-3.32)^***^
Family members	Absence	41.1 (841/2,045)	1.0	1.0	1.0
	Presence	49.2 (547/1,112)	1.43 (1.23-1.66)	1.43 (1.23-1.66)	1.29 (1.10-1.50)^****^

Response variable: 1 = participation group and 0 = nonparticipation group. ^*^Model 1: Adjusted for age, sex, education level (≤12 or >12 years), subjective economic status (very good plus good, average, or poor plus very poor), occupation (self-employed workers, part-time workers, plus farmers, homemakers, or unemployed plus retired), smoking status (current smokers, ex-smokers, or nonsmokers), body mass index (≥25.0 or <25.0 kg/m^2^), and regular visits to medical institutions (presence or absence). ^**^Adjusted for all factors in Model 1 plus recommendation from primary care physicians (presence or absence) and recommendation from family members (presence or absence). ^***^Adjusted for all factors in Model 1 plus recommendation from public health nurses (presence or absence) and recommendation from family members (presence or absence). ^****^Adjusted for all factors in Model 1 plus recommendation from public health nurses (presence or absence) and recommendation from primary care physicians (presence or absence). OR, odds ratio; CI, confidence interval.

**Table 4.** Multivariable-adjusted Odds Ratios and 95% Confidence Intervals for Participation in Specific Health Checkups by Recommendations from Public Health Nurses, Primary Care Physicians, Nurses, and Family Members among Middle-aged Community Residents with National Health Insurance: Stratified Analysis by Regular Visits to Medical Institutions.

Recommendation from public health nurses, medical professionals, and family members to participate in specific health checkups in the past year	Comparison	Proportions of those in the participation group, % (case/n)	Age-adjusted OR (95% CI) for participation in specific health checkups	Multivariable-adjusted OR (95% CI) for participation in specific health checkups	
				Model 1^*^	Model 2
Community residents with regular visits to medical institutions (n = 7,509)					
Public health nurses	Absence	42.3 (3,057/7,231)	1.0	1.0	1.0
	Presence	57.6 (160/278)	1.81 (1.42-2.31)	1.83 (1.43-2.34)	1.63 (1.26-2.09)^**^
Primary care physicians	Absence	37.8 (2,303/6,093)	1.0	1.0	1.0
	Presence	64.5 (914/1,416)	2.92 (2.58-3.29)	3.02 (2.67-3.42)	2.92 (2.58-3.31)^***^
Nurses	Absence	42.3 (3,092/7,308)	1.0	1.0	1.0
	Presence	62.2 (125/201)	2.28 (1.71-3.05)	2.44 (1.82-3.27)	2.22 (1.65-2.99)^***^
Family members	Absence	40.6 (1,980/4,876)	1.0	1.0	1.0
	Presence	47.0 (1,237/2,633)	1.34 (1.22-1.47)	1.32 (1.20-1.45)	1.19 (1.08-1.32)^****^
Community residents with irregular visits to medical institutions (n = 3,671)					
Public health nurses	Absence	31.1 (1,103/3,551)	1.0	1.0	1.0
	Presence	53.3 (64/120)	2.49 (1.73-3.59)	2.54 (1.75-3.70)	2.34 (1.60-3.43)^**^
Primary care physicians^†^	Absence	37.6 (789/2,101)	1.0	1.0	1.0
	Presence	58.8 (104/177)	2.31 (1.69-3.16)	2.31 (1.68-3.18)	2.11 (1.53-2.91)^***^
Nurses^†^	Absence	39.0 (877/2,251)	1.0	1.0	1.0
	Presence	59.3 (16/27)	2.26 (1.04-4.90)	2.40 (1.09-5.29)	2.22 (1.002-4.93)^***^
Family members	Absence	29.4 (624/2,126)	1.0	1.0	1.0
	Presence	35.1 (543/1,545)	1.33 (1.16-1.53)	1.34 (1.16-1.55)	1.28 (1.11-1.49)^****^

Response variable: 1 = participation group and 0 = nonparticipation group. ^*^Model 1: Adjusted for age, sex, education level (≤12 or >12 years), subjective economic status (very good plus good, average, or poor plus very poor), occupation (self-employed workers, part-time workers, plus farmers, homemakers, or unemployed plus retired), stages of health behavior change (precontemplation, contemplation plus preparation, or action plus maintenance), smoking status (current smokers, ex-smokers, or nonsmokers), and body mass index (≥25.0 or <25.0 kg/m^2^). ^**^Adjusted for all factors in Model 1 plus recommendation from primary care physicians (presence or absence) and recommendation from family members (presence or absence). ^***^Adjusted for all factors in Model 1 plus recommendation from public health nurses (presence or absence) and recommendation from family members (presence or absence). ^****^Adjusted for all factors in Model 1 plus recommendation from public health nurses (presence or absence) and recommendation from primary care physicians (presence or absence). ^†^Excluded 893 community residents who visited medical institutions 0 times in the past year. OR, odds ratio; CI, confidence interval.

Of 11,180 community residents, 12.7%, 65.4%, 18.8%, and 53.8% had needs for recommendation from public health nurses, primary care physicians, nurses, and family members, and 3.6%, 14.6%, 2.2%, and 37.4% were recommended from these sources in the past year (Table 5).

**Table 5.** Proportions of Those Who Received Recommendations from Public Health Nurses, Medical Professionals, and Family Members and Those Who Had Needs for Recommendations.

	Proportions of those who received recommendations from public health nurses, medical professionals, and family members in the past year^*^	Proportions of those who had needs for recommendations from public health nurses, medical professionals, and family members^†^
n	11,180	11,180
Public health nurses, n (%)	398 (3.6)	1,425 (12.7)
Primary care physicians, n (%)	1,627 (14.6)	7,311 (65.4)
Nurses, n (%)	242 (2.2)	2,106 (18.8)
Family members, n (%)	4,178 (37.4)	6,012 (53.8)

^*^ “Did public health nurses, primary care physicians, nurses, and family members recommended that you undergo a specific health checkup in the past year?” ^†^ “From whom would you like to receive a recommendation to undergo a specific health checkup?”

## Discussion

This large-scale, community-based study clearly showed that after adjusting for confounding factors, the presence of recommendations from public health nurses, medical professionals (primary care physicians and nurses), and family members was positively associated with participation in specific health checkups in this Japanese population. This study highlighted the importance of recommendations from medical professionals at medical institutions and from family members at home as well as from municipal public health nurses. Notably, a previous study regarding cancer screening behavior reported that people sought recommendations from reliable sources ^(14)^. The present study also demonstrated the strong impact of recommendations from medical professionals, especially primary care physicians.

In this study, those who had received a recommendation from primary care physicians in the past year were approximately 2.8 times more likely to have undergone a specific health checkup in the past year than those who had not received a recommendation. In addition, regardless of the stage of health behavior change or regular visits to medical institutions, the presence of recommendations from primary care physicians was positively associated with participation in specific health checkups. In a previous study involving Asians, recommendations from primary care physicians were reported to be the most effective way to promote colorectal cancer screening behavior ^(8)^. Therefore, recommendations from primary care physicians may be an important factor in promoting health checkup behavior. Notably, the proportion of those who had needs for recommendations from primary care physicians was high (65.4%), but the proportion of those who had received a recommendation from primary care physicians was low (14.6%). Therefore, a system that promotes recommendations from primary care physicians at medical institutions may be necessary.

We found that those who had received recommendations from nurses in the past year were approximately 2.1 times more likely to have undergone a specific health checkup in the past year than those who had not received recommendations. Moreover, the stratified analysis showed that recommendations from nurses were positively associated with participation in health checkups, except for among participants in the precontemplation stage. Previous studies reported the effect of recommendations from primary care physicians on cancer screening behavior ^(8), (9), (10)^. However, no studies reported the effect of recommendations from nurses. Therefore, this study clarified for the first time the effect of recommendations from nurses on checkup behavior. This suggested that recommendations from both primary care physicians and nurses are important at medical institutions. For community residents who regularly visit medical institutions, it may be effective for nurses to proactively recommend specific health checkups during the waiting time at the medical institutions.

In this study, those who received recommendations from public health nurses in the past year were approximately 1.8 times more likely to have undergone specific health checkups in the past year than those who had not received recommendations. In addition, regardless of the stages of health behavior change or regular visits to medical institutions, the presence of recommendations from public health nurses was positively associated with participation in specific health checkups. A previous study reported that telephone recommendations were effective in cancer screening behavior ^(15)^. Therefore, recommendations from public health nurses via telephone may be important, especially for community residents without regular visits to medical institutions. However, the proportion of those who had received recommendations from public health nurses was low (3.6%). This suggests that increasing opportunities for community residents to receive recommendations from public health nurses are also a challenge.

Our findings showed that those who received recommendations from family members in the past year were approximately 1.2 times more likely to have undergone specific health checkups in the past year than those who had not received recommendations. Moreover, regardless of the stage of health behavior change or regular visits to medical institutions, the presence of recommendations from family members was positively associated with participation in specific health checkups. Although the OR for participation in specific health checkups was 1.2, which was lower than the ORs for medical professionals and public health nurses, the proportion of those who had received recommendations for specific health checkups from family members was the highest (37.4%). Therefore, recommendations from family members are as important as recommendations from medical professionals and public health nurses.

The strength of the present study was the inclusion of a large sample of community residents with NHI aged 40-64 years from five cities in Osaka Prefecture, Japan. However, this study also had several limitations. First, a cross-sectional design cannot prove causality. Therefore, a prospective study is necessary to confirm our findings. Second, we cannot exclude the possibility of selection bias because the response rate was 36.7%. Third, in our study, data for recommendations from public health nurses, medical professionals, and family members as well as data for participation in specific health checkups were assessed by self-report. Therefore, we cannot exclude the possibility of recall bias. Those who participated in health checkups may have been more likely to remember recommendations from public health nurses, medical professionals, and family members. To reduce recall bias, our questionnaire asked regarding recommendations from public health nurses, medical professionals, and family members in the past year (presence or absence) and the need for recommendations from public health nurses, medical professionals, and family members (presence or absence) in Q1. Items covering participation in specific health checkups in the past year (presence or absence) were included in a later section. However, a properly designed intervention study is necessary to confirm our findings. Finally, because this study used a questionnaire that asked regarding the presence or absence of recommendations from public health nurses, medical professionals (primary care physicians and nurses), and family members regarding participation in specific health checkups in the past year, we did not assess the content of these recommendations. Despite these limitations, the present findings support the conclusion that the presence of recommendations from public health nurses, medical professionals (primary care physicians and nurses), and family members may be important to promote participation in specific health checkups among middle-aged Japanese community residents with NHI.

## Article Information

### Conflicts of Interest

None

### Sources of Funding

This work was supported by the Fund for Health Promotion from Osaka prefecture.

### Acknowledgement

We are grateful to all participants who took part in this research. We thank Kyoko Tanaka, Maiko Shikama, Nao Fujiyama and all members of this project. We thank Hiroyasu Iso and Hideo Tanaka for their advice on this project.

### Author Contributions

NA: conceptualization, data curation, analysis, funding acquisition, investigation, project administration, and writing-original draft. CK, RY, KW, and CT: investigation, project administration, and review and editing. ML: data curation, investigation, and review and editing. AM: conceptualization, data curation, funding acquisition, investigation, project administration, and review and editing.

### Approval by Institutional Review Board (IRB)

Approval code issued by the Institutional Review Boards: 2020-28.

The name of the Institutions: Osaka Prefecture University

The study protocol was prepared in accordance with the Declaration of Helsinki.

### Data Availability

Data cannot be shared for privacy or ethical reasons.
